# A brief historical perspective on cell cycle control of CENP-A assembly and inheritance

**DOI:** 10.1007/s10577-025-09774-2

**Published:** 2025-07-26

**Authors:** Grant Rowley, Lars E. T. Jansen

**Affiliations:** Department of Biochemistry, University of Oxford, Oxford, OX1 3QU UK

**Keywords:** Centromere, Epigenetics, CENP-A, Mis18 complex, HJURP, Phosphoregulation

## Abstract

Centromeres provide the chromosomal scaffold for the assembly of the kinetochore complex, thereby linking replicated sister chromatids to the mitotic spindle, driving their segregation into nascent daughter cells. The location and maintenance of centromeres rely, in large part, on a unique conserved chromatin domain, defined by nucleosomes containing the histone H3 variant, Centromere Protein A (CENP-A), whose discovery 40 years ago we now celebrate. Current models place CENP-A, along with many of its orthologs, at the centre of a self-propagating epigenetic feedback loop that heritably maintains centromere position through mitotic and meiotic divisions. CENP-A is stably recycled through DNA replication but requires replenishment each cell cycle. In many organisms, assembly is restricted to G1 phase, indicating tight cell cycle control of the assembly machinery. Here, we provide a historical overview of the discoveries that led to current models of cell cycle control of centromere assembly, starting with early models of regulation to the intricate, multi-layered phosphoregulation revealed to date. Our review focuses primarily on the human and other animal systems, in which the current view is that negative and positive control through cyclin-dependent kinases and Polo-like kinase 1 combine to link CENP-A assembly to mitotic exit. Cell cycle-coupled CENP-A assembly has been attributed to so-called licensing or priming events. We discuss the validity of these models and terminology and highlight key outstanding questions that remain unanswered.

## Molecular definition and epigenetic nature of human centromeres

Among the earliest descriptions of chromosomes are those by Walther Flemming who revealed a peculiar feature, the “primary constriction” (Flemming 1882). This chromosomal domain, later named the"centromere"(Darlington and Hall 1936) was recognised by early geneticists as the site of suppressed meiotic recombination and, importantly, the locus on the chromosome essential for its inheritance. By the 1970s, satellite DNA, short homogeneously repeated sequences discovered in the early sixties (Kit 1961), were found to map to centromeres and pericentric heterochromatic regions (Pardue and Gall 1970). At human centromeres, these sequences are made up of α-satellites, consisting of hundreds of kilobases of AT-rich repeats (Wevrick and Willard 1989).

EM studies in the 1960s defined a multilayered structure consisting of a proteinaceous chromosomal proximal region (the centromere) and a distal region (the kinetochore) composed of components interacting with microtubules, connecting chromosomes to the mitotic spindle (Brinkley and Stubblefield 1966). However, the molecular composition remained unknown until advances in molecular biology in the 1980s. In 1985, Earnshaw and Rothfield made the initial breakthrough in identifying the molecular composition of the human centromere. Using sera isolated from patients presenting with scleroderma (CREST), they identified antigens that were resolved as three distinct polypeptides localising to human centromeres: CENP-A, CENP-B, and CENP-C (Earnshaw and Rothfield 1985). The cloning of CENP-B and the discovery of its binding to α-satellite DNA implied a key function of DNA sequence in specifying centromeres (Earnshaw et al. 1987). However, observations by Earnshaw and Migeon revealed that centromeres can be inactivated despite the presence of α-satellites (Earnshaw and Migeon 1985). This was an early indication that the underlying DNA sequence is insufficient to maintain centromere function.

Moreover, the subsequent discovery by Andy Choo’s lab of a functional centromere arising de novo on a region lacking any α-satellites or CENP-B showed that centromeres can function, at least in some instances, independently of α-satellite DNA (Voullaire et al. 1993; Warburton et al. 1997; Amor et al. 2004; Marshall et al. 2008). The existence of so-called neocentromeres indicates there must be another α-satellite-independent mechanism that specifies the location of the centromere. Histone proteins are an ideal candidate for such a role, as they bind DNA stably yet in a non-sequence-specific manner.

### Evidence for CENP-A as the defining mark of the centromere

CENP-A was initially found to copurify with nucleosomal particles and feature a histone H3-like domain, strongly suggesting that CENP-A is a centromere-specific chromatin component (Palmer et al. 1987, 1991). The cloning of a human CENP-A cDNA revealed that it is a variant of histone H3 (Sullivan et al. 1994). Remarkably, CENP-A was found to be retained in mature bovine sperm, an early indicator of CENP-A's role in centromere inheritance, possibly even across generations (Palmer et al. 1990), for which later direct functional evidence was found in Drosophila (Raychaudhuri et al. 2012). In addition, CENP-A was found to be conserved across disparate eukaryotes and essential for centromere function (Stoler et al. 1995; Henikoff et al. 2000; Howman et al. 2000; Régnier et al. 2005). Early structural studies identified a divergent centromere targeting domain (CATD) within the H3-like histone core composed of a divergent loop 1 and α2-helix. This domain directs CENP-A to the centromere, conveys rigidity to CENP-A nucleosomes and interacts with CENP-N (Black et al. 2004, 2007; Carroll et al. 2009).

A key insight into the central role of CENP-A in defining centromeres came from the ectopic recruitment of CENP-A to naïve, non-centromere loci in *Drosophila*, either by overexpression (Heun et al. 2006; Olszak et al. 2011) or by directed targeting to a LacO array (Mendiburo et al. 2011), leading to the formation of a functional centromere, capable of accurately directing chromosome segregation. Crucially, once formed ectopically, centromere maintenance is no longer dependent on the initial seed, demonstrating a classic epigenetic mode of inheritance where CENP-A is the critical seed that defines the chromosomal locus of the centromere (Jansen 2012).

**Fig. 1:**
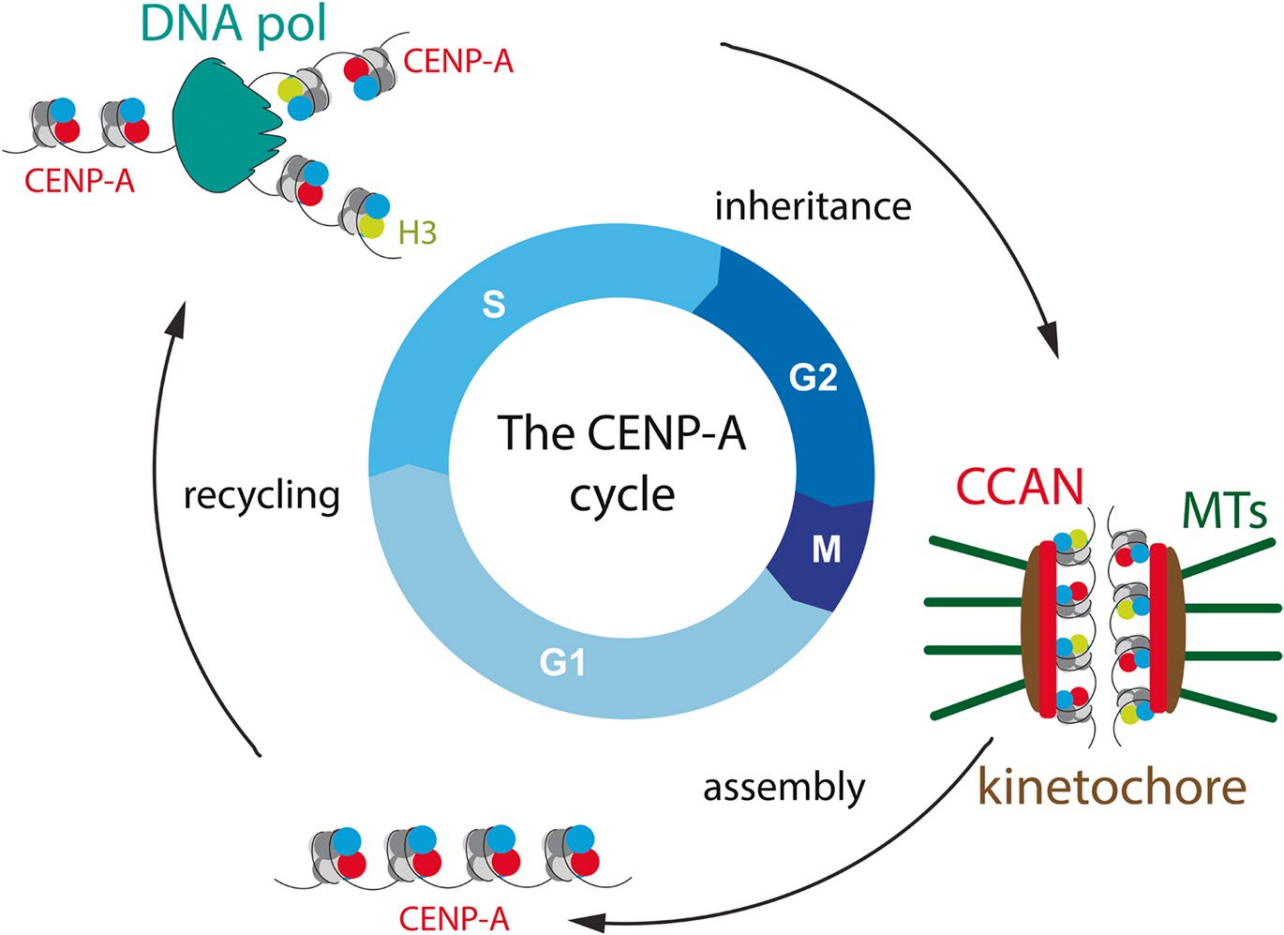
The fundamental logic of the CENP-A cycle in human cells. CENP-A assembly is strictly limited to G1 phase and is subsequently stably retained in chromatin. Consequently, CENP-A is diluted in S phase, distributed onto the two sister chromatids during H3 nucleosome assembly, reducing the CENP-A complement per centromere in half. This CENP-A/H3 nucleosome mixed state forms the functional substrate for kinetochore assembly in mitosis and a template for new CENP-A assembly in the next cell cycle. DNA pol: DNA polymerase. CCAN: Constitutive Centromere-Associated Network. MTs: Microtubules

The essential role of CENP-A chromatin for initiating centromere formation and maintaining centromere function suggests CENP-A acts as the epigenetic determinant of the centromere. One key property required of such a “mark” is stable transmission through mitotic and even meiotic divisions. Indeed, CENP-A nucleosomes are retained at centromeres more efficiently than canonical H3 nucleosomes. Fluorescent pulse labelling (Jansen et al. 2007; Bodor et al. 2013) and FRAP studies (Hemmerich et al. 2008) revealed a lack of significant turnover of CENP-A nucleosomes and stable transmission of CENP-A protein through cell division cycles (Mitra et al. 2020b) (Fig. 1). This retention of ancestral CENP-A molecules in chromatin is consistent with the transmission of CENP-A chromatin through mitotic cell divisions, and even through the germline (Palmer et al. 1990; Smoak et al. 2016; Das et al. 2023). This behaviour contrasts with other centromere-associated proteins, which associate more dynamically (Hemmerich et al. 2008; Prendergast et al. 2016; Watanabe et al. 2022), and is consistent with a central role for CENP-A in the heritable maintenance of centromere position and function in both the soma and germline.

### CENP-A assembly is uncoupled from DNA synthesis but linked to mitotic exit

The emerging role of CENP-A in maintaining centromere identity raises the acute question of how CENP-A chromatin is assembled and transmitted. The initial default hypothesis would be that CENP-A is incorporated alongside canonical histones during DNA replication. However, early work at the turn of the century by Shelby and Sullivan showed that CENP-A expression peaks in G2 and that forced expression of CENP-A in the S phase results in its mislocalisation (Shelby et al. 1997; Sullivan 2001). Moreover, centromere targeting of CENP-A occurs independently of DNA synthesis in flies and human cells (Shelby et al. 2000; Ahmad and Henikoff 2001).

This led to the surprising realisation that CENP-A deposition is uncoupled from canonical replication-dependent histone assembly. Early models attempting to explain how CENP-A incorporation could be linked to the cell cycle included the timing of its expression (Shelby et al. 1997) or the uncoupling of replication of centromeres from the rest of the genome, thereby creating distinct spatial or temporal windows for centromeric chromatin assembly (Csink and Henikoff 1998; Ahmad and Henikoff 2001). However, these early models were inconsistent with the asynchronous and variable replication timing of distinct centromeres (Shelby et al. 1997; Sullivan and Karpen 2001).

Quantitative fluorescence microscopy and photobleaching in *Drosophila* embryos (Schuh et al. 2007), as well as fluorescent quench-chase pulse labelling using the then emerging SNAP-tag technology in human cells (Jansen et al. 2007), demonstrated that CENP-A assembly is tightly coupled to exit from mitosis (Fig. 1). FRAP studies confirmed that CENP-A is dynamic in G1 but stable in chromatin for the remainder of the cell cycle, consistent with a G1 phase-specific assembly pathway (Hemmerich et al. 2008).

### The discovery of CENP-A assembly machinery and its temporal localisation

The discovery of cell cycle-restricted loading of CENP-A coincided with the identification of key proteins driving CENP-A nucleosome assembly in the mid-2000s. Missegregation (*mis*) mutants in fission yeast identified the first proteins required for CENP-A assembly, including Mis18, which is essential for CENP-A localisation in yeast (Takahashi et al. 2000; Hayashi et al. 2004). Mis18 has two human homologs, Mis18α and Mis18β, which play a similarly critical role (Fujita et al. 2007). Both proteins, along with an additional binding partner, M18BP1 (Fujita et al. 2007; Maddox et al. 2007), localise to human centromeres exclusively in anaphase and the first few hours of early G1 phase and are essential for the deposition of new CENP-A.

Furthermore, CENP-A chromatin purification from human cells led to the discovery of a set of associated protein subcomplexes (Foltz et al. 2006; Okada et al. 2006; Izuta et al. 2006), later named the “Constitutive Centromere-Associated Network (CCAN)” (Cheeseman and Desai 2008), which forms the platform for assembly of the microtubule-binding complexes of the kinetochore. One inconspicuous co-purifying component identified by Foltz and colleagues was FLEG1, renamed a year later as Holliday Junction Recognition Protein (HJURP) after being characterised as a protein involved in DNA damage repair (Kato et al. 2007). This enigmatic role remains unconfirmed and has yet to be fully investigated. While HJURP was found to only weakly associate with CENP-A chromatin, purification of pre-nucleosomal CENP-A identified HJURP as a stoichiometric CENP-A-specific chaperone. It binds the CENP-A centromere-targeting domain (CATD), localises to the centromere in telophase/early G1 and is required for the deposition of nascent CENP-A at centromeres (Dunleavy et al. 2009; Foltz et al. 2009). Similarly, work in yeasts and *Drosophila* identified Scm3 (Mizuguchi et al. 2007; Stoler et al. 2007; Camahort et al. 2007; Pidoux et al. 2009; Sanchez-Pulido et al. 2009) and CAL1 (Goshima et al. 2007; Erhardt et al. 2008; Mellone et al. 2011), respectively, as the equivalent CENP-A chaperone.

Crucially, tethering HJURP or CAL1 to a naïve chromosomal locus (via a LacO array) is sufficient to nucleate CENP-A chromatin, resulting in kinetochore recruitment and attachment to microtubules (Barnhart et al. 2011; Chen et al. 2014). Importantly, in both studies, once centromeres were established, their maintenance became independent of the LacI-seed. These studies underscored the central role of these chaperones in CENP-A assembly and the epigenetic definition of the centromere.

### Cyclin-dependent kinases restrict CENP-A assembly to early G1 phase by preventing Mis18 complex formation

**Fig. 2:**
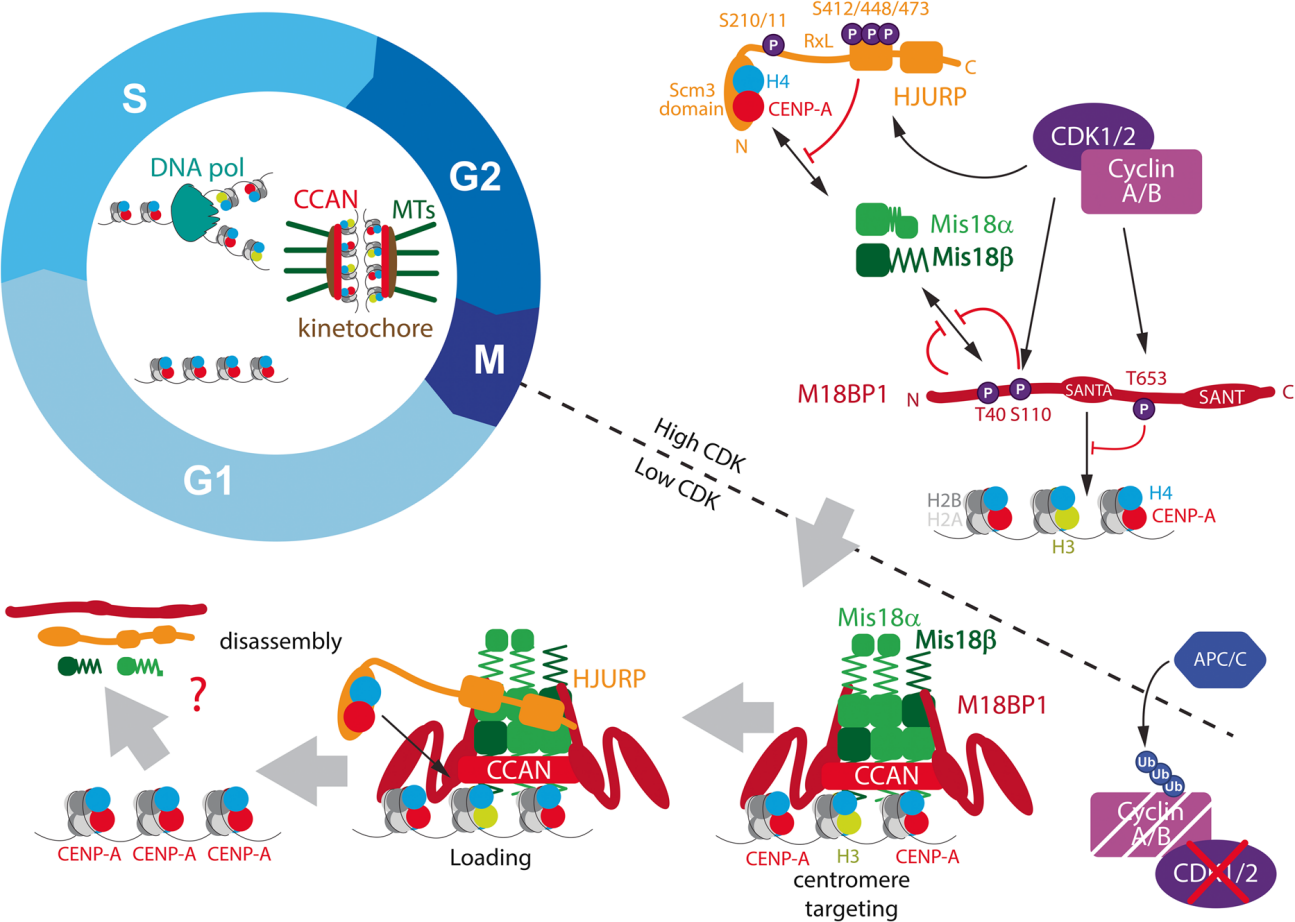
Negative phosphoregulation restricts CENP-A assembly to mitotic exit. The CENP-A assembly factors M18BP1 (red), Mis18α and β (green), and the CENP-A chaperone HJURP (orange) are expressed throughout the cell cycle. High CDK2 and CDK1 activity in S, G2, and mitosis phosphorylates M18BP1 and HJURP at key residues, preventing M18BP1/Mis18α/β complex formation and the targeting of this complex and HJURP to centromeres. Loss of mitotic CDK activity during anaphase results in the rapid loss of inhibitory phosphorylation, facilitating assembly factor complex formation and centromere targeting, enabling CENP-A loading in the G1 phase. Key proteins, protein domains and phospho-sites are indicated

The targeting of the Mis18 complex and HJURP to centromeres during a unique temporal window at mitotic exit strongly suggests that these factors are under cell cycle control. An early insight came from treating cells with cyclin-dependent kinase (CDK) inhibitors. These experiments were initially intended to uncouple CENP-A loading in G1 phase from normal mitotic progression, but resulted in the serendipitous discovery of CDK-mediated phosphoregulation as a key controller of CENP-A assembly (Silva et al. 2012). Conditional mutations in DT40 cells identified CDK1 and CDK2 as inhibitors of CENP-A assembly, and M18BP1 was found to be phosphorylated in a CDK-dependent manner (Silva et al. 2012), preventing its centromere targeting. Subsequent studies identified Threonine-653 (T653) as a crucial CDK substrate, and mutation of this residue alone was sufficient to constitutively localise M18BP1 to centromeres (Stankovic et al. 2017)(Fig. 2).

Biochemical studies of M18BP1 uncovered the stoichiometry of the Mis18 complex as a Mis18α:Mis18β 4:2 hexamer that is bound to the M18BP1 N-terminal domain (Pan et al. 2017; Spiller et al. 2017) (Fig. 2). This interaction is prevented by CDK phosphorylation of T40, and S110 residues within the M18BP1 N-terminal domain, restricting recruitment of the Mis18α:Mis18β hexamer to early G1 when CDK activity drops (Fig. 2).

The binding partners recruiting the Mis18 complex to the centromere are not well defined. M18BP1 likely interacts with CENP-C (Moree et al. 2011; Dambacher et al. 2012; Shono et al. 2015; French and Straight 2019).

Furthermore, in Xenopus extract and chicken DT40 cells, M18BP1 can directly interact with CENP-A nucleosomes through a CENP-C-like motif (French et al. 2017; Hori et al. 2017), which is responsible in part for M18BP1 localisation to centromeres. This domain is absent from human M18BP1, indicating that other interaction surfaces, including CENP-C, are required for robust Mis18 complex recruitment. However, how M18BP1 phosphorylation disrupts centromere recognition remains an important open question.

### Mitotic CDK activity prevents HJURP recruitment and loading of new CENP-A

Anaphase targeting of the Mis18 complex precedes the arrival of HJURP and is required for its recruitment to the centromere. Analogous to M18BP1, HJURP is also a CDK target and interacts with cyclin A through an RxL motif in a vertebrate-conserved domain (Stankovic et al. 2017)(Fig. 2). Genetic disruption of this domain or CDK target residues in the C-terminal domain of HJURP results in unscheduled centromere localisation throughout the cell cycle (Müller et al. 2014; Stankovic et al. 2017). Crucially, forced expression of M18BP1 mutated at T653 and HJURP mutated at its cyclin A interaction motif reconstitute G1-like levels of CENP-A loading before mitosis (Stankovic et al. 2017). This highlights that these two protein complexes are likely the principal targets of CDK-mediated negative control that is relieved during anaphase, triggering centromere recruitment and the loading of new CENP-A nucleosomes (Fig. 2).

The C-terminal R1 and R2 domains of HJURP were found to be critical for binding to Mis18α (Nardi et al. 2016; Pan et al. 2019). This domain is indispensable for the localisation of HJURP and is dotted with CDK sites (Müller et al. 2014; Stankovic et al. 2017), which may negatively regulate this interaction, although this remains to be tested. These results indicate that high CDK activity in S, G2, and mitosis prevents CENP-A assembly by preventing centromere localisation of the Mis18 complex and by disrupting downstream HJURP recruitment.

### PLK1 phosphorylation facilitates assembly of CENP-A by stabilising the HJURP-Mis18 interaction

**Fig. 3:**
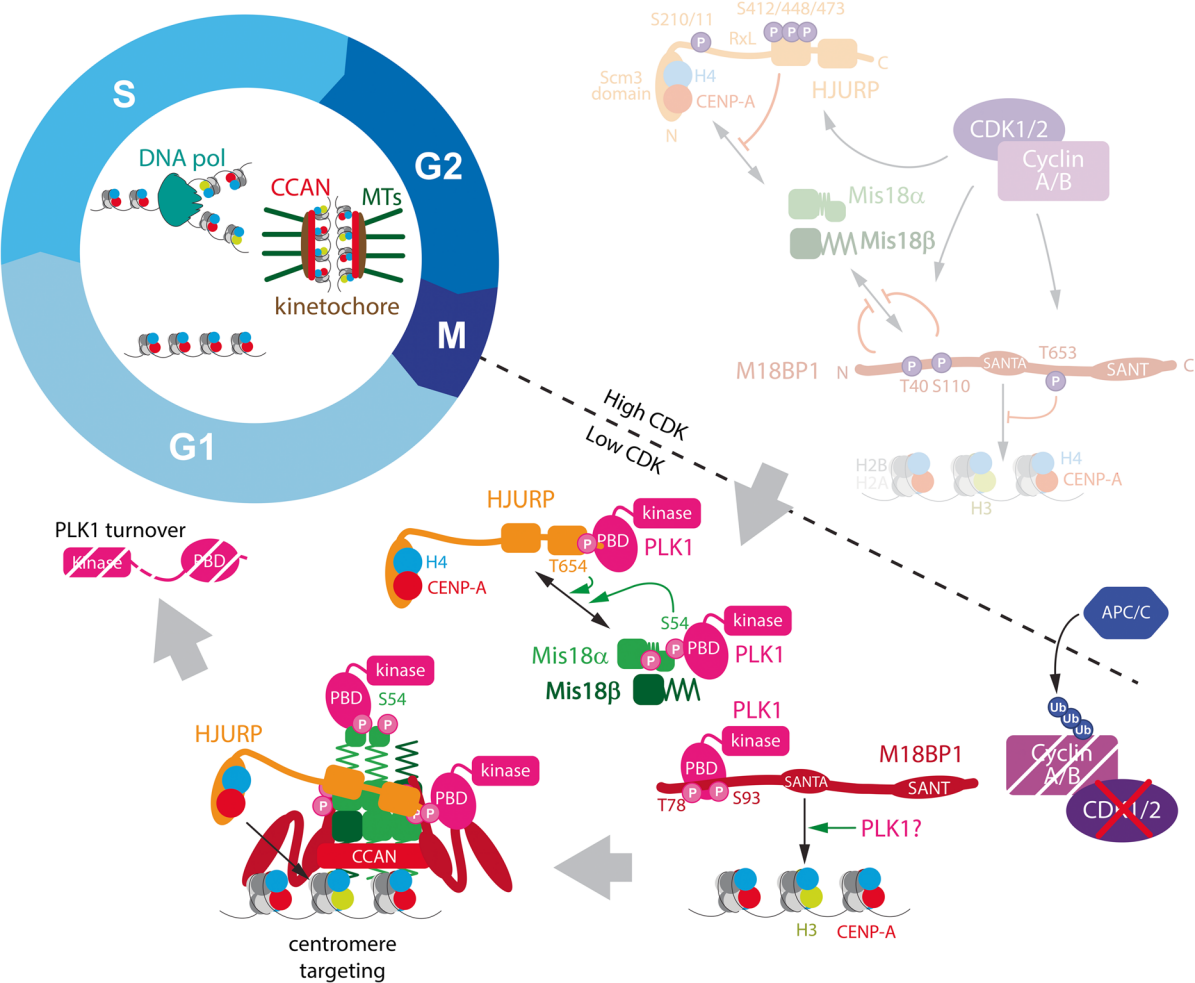
Release of negative phosphoregulation coincides with positive phosphoregulation driving CENP-A assembly in the G1 phase. Upon release from pre-mitotic inhibition (dimmed in figure) in anaphase, the mitotic kinase PLK1 binds and phosphorylates key residues in Mis18BP1 that facilitate further stable PLK1 binding through its polo box domain (PBD) to the Mis18 complex. PLK1 binding and phosphorylation of the N-terminal alpha helix in Mis18α (Ser 54) allosterically release the inhibitory alpha helix (zig zag), driving HJURP binding to the Mis18 complex. PLK1 may also phosphorylate additional sites that target Mis18BP1 to the centromere

In addition to the negative phosphoregulation by CDKs, a second positive form of regulation was discovered by McKinley and Cheeseman, who showed that Polo-like kinase 1 (PLK1) activity is required for the assembly of new CENP-A (McKinley and Cheeseman 2014). PLK1 binds to and phosphorylates M18BP1 in G1 phase. Mutational analysis identified key PLK1 target residues in the N-terminus of M18BP1 whose phosphorylation does not impact Mis18 complex formation per se*,* but rather promotes M18BP1 localisation to the centromere and the downstream recruitment of HJURP (Fig. 3).

More recent work further defined PLK1 action. Following the initial binding of PLK1 to the N-terminus of M18BP1, depending on Threonine-78 and Serine-93, it phosphorylates Serine-54 on Mis18α and Threonine-654 on HJURP (Fig. 3). Interestingly, these phospho-sites are self-priming, acting as a docking site for the formation of a stable PLK1-Mis18 complex via its Polo-box binding domain (Conti et al. 2024; Parashara et al. 2024). This facilitates the release of Mis18α’s N-terminal domain from an inhibitory conformation, allowing the binding of HJURP to the complex, crucial for the loading of new CENP-A (Conti et al. 2024; Parashara et al. 2024). Whilst the latter studies identified a clear PLK1-dependent mechanism for HJURP recruitment, the initial McKinley study showed that upon global PLK1 inhibition, initial centromere recruitment of the Mis18 complex is also impaired. This suggests that PLK1 could also promote earlier steps in Mis18 complex targeting to the centromere, although indirect effects of the inhibitor cannot be excluded.

Combined, these studies demonstrate that opposing PLK1 and CDK1/2 kinase activities act as a two-factor authentication mechanism, likely defining the tight temporal control of CENP-A assembly. The strict regulation of the assembly factors by PLK1 may further restrict the timing of CENP-A deposition, as PLK1, similarly to cyclins, is degraded upon mitotic exit (Lindon and Pines 2004). This results in a brief window of opportunity where CENP-A loading occurs after Cyclin B destruction and CDK1 inactivation but before PLK1 degradation. The loss of PLK1 activity and concurrent action of phosphatases may result in the disassembly of the CENP-A deposition machinery, which has been observed to occur in mid-G1 phase (Fig. 2)(Fujita et al. 2007; Dunleavy et al. 2009; Foltz et al. 2009). However, the mechanisms that terminate CENP-A assembly and whether timely termination is important for centromere integrity remain largely unknown. In this context, an interesting observation is that assembly of CENP-A is linked to the dissociation of M18BP1 from the centromere, which could be part of a mechanism to limit assembly (Stankovic et al. 2017).

### The concept of licensing CENP-A assembly

The temporal uncoupling of CENP-A assembly in G1 phase from the replication of underlying DNA in S phase draws interesting parallels to the “licensing” of DNA replication. Here, licensing of origins in G1 permits the execution of DNA replication in the S phase. Central to the concept of licensing is that no replication can occur during the licensing stage, and conversely, no re-licensing can occur once replication has commenced. This ensures that the execution of DNA replication critically depends on progression through both temporally distinct cell cycle stages, thereby limiting replication to only once per cycle.

Analogously, CENP-A loading is tightly regulated, occurs only once per cell cycle, and coincides with the timing of DNA replication licensing. While the terms"licensing"and"priming"are both used in the CENP-A assembly lexicon, there is, thus far, no strong evidence of actual licencing of CENP-A assembly, at least not in a manner analogous to the licencing of DNA replication, where a mitotic or pre-mitotic event signals for CENP-A assembly upon mitotic exit.

It was initially proposed that Mis18 proteins “license” the centromere in G1, possibly through an acetylation mechanism, for CENP-A assembly later in the cell cycle (Fujita et al. 2007). At the time, CENP-A deposition was assumed to occur post-S phase (Shelby et al. 2000). This suggested the deposition of a Mis18-dependent “licensing” mark in early G1, used later in the cell cycle to trigger CENP-A assembly (Fujita et al. 2007). It is an important historical nuance that this proposition was made just weeks before the discovery that CENP-A assembly, in fact, occurs simultaneously with Mis18 centromere targeting (Schuh et al. 2007; Jansen et al. 2007). Thus, the notion of licensing chromatin for a later deposition step proved irrelevant in this context. While the term continues to be used in the literature [e.g. (McKinley and Cheeseman 2014; Nardi et al. 2016; Pan et al. 2019; Conti et al. 2024; Parashara et al. 2024)], its meaning requires a more careful definition.

The term “priming” may agree better with current evidence of CENP-A assembly control, a term used in the context of PLK1 action in CENP-A assembly (McKinley and Cheeseman 2014; Conti et al. 2024; Parashara et al. 2024). Here, priming refers specifically to PLK1 generating its own binding sites through phosphorylation, thereby priming downstream events (Fig. 3). However, these events are not separated in time and thus far, not known to be dependent on prior licensing events.

The initial implication of an acetylation step in Mis18 action has remained largely unexplored. However, intriguingly, tethering of histone acetyltransferases may partially bypass the requirement for Mis18α for CENP-A assembly (Ohzeki et al. 2012). Thus, while mechanisms remain unknown, there appears to be a tangible link between acetylation and Mis18 action.

### What would constitute true licensing?

Current evidence in human cells suggests that cells only need to “be” in early G1 phase to load CENP-A. To license CENP-A assembly, one would envision an exclusively mitotic or pre-mitotic event that is required to assemble CENP-A in the subsequent G1.

**Fig. 4:**
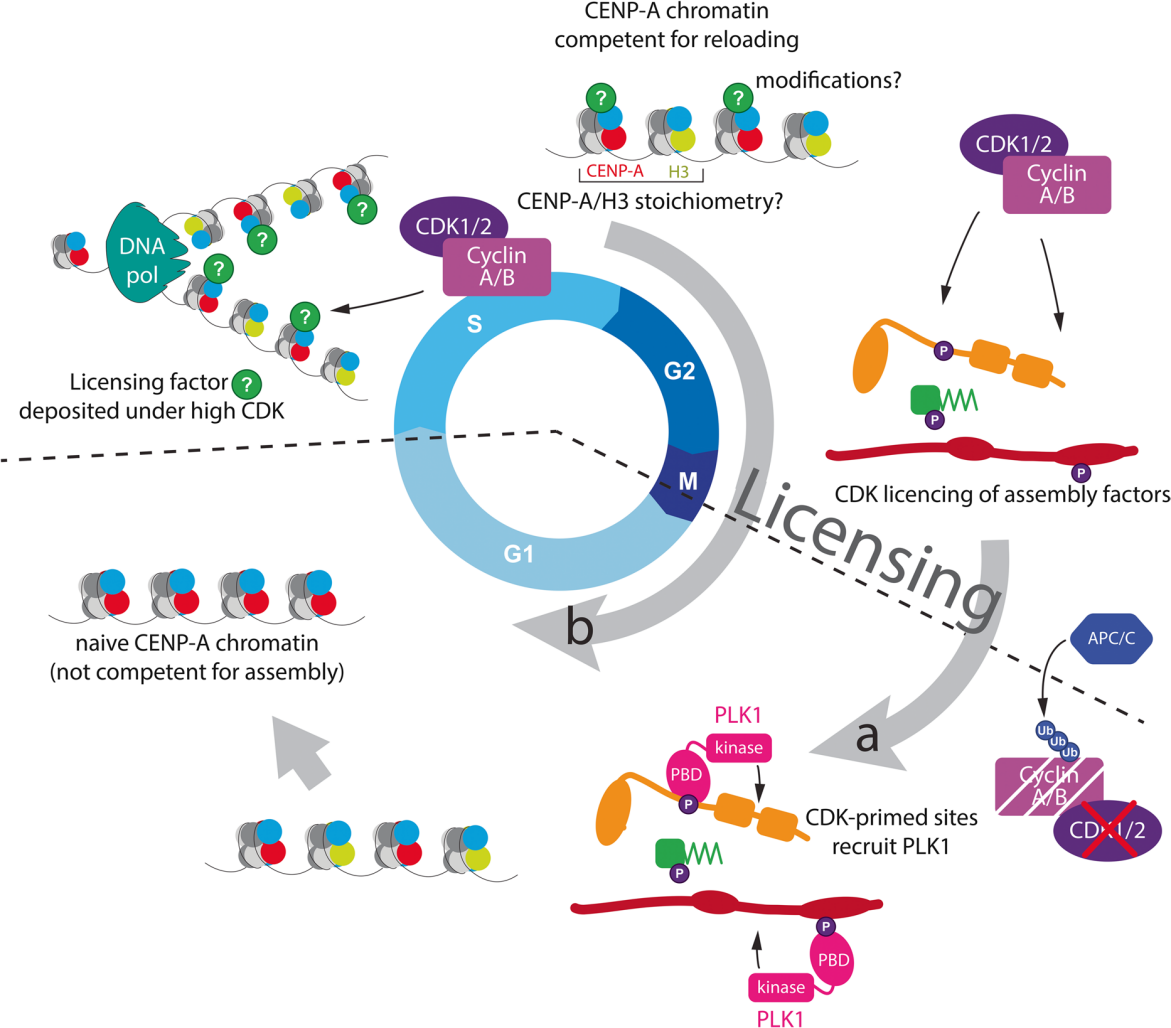
Possible mechanisms of S-G2-M phase licensing of subsequent CENP-A assembly in G1 phase. a) Pre-mitotic licensing may occur at the level of phospho-control. One plausible mechanism includes CDK-mediated licensing of assembly factors that “prime” the binding of PLK1 in G1 phase to trigger CENP-A assembly factor complex formation and centromere targeting (grey arrow “a”). In this way, CDK1/2-driven events in S, G2, and M are essential to “license” G1 phase assembly of centromeric chromatin. b) Newly assembled CENP-A nucleosomes may be poor substrates for templating the assembly machinery. Naïve CENP-A chromatin may be “licensed” in S, G2, or M phases through a CDK1/2-dependent step. This may include binding of “maturation” factors (green?), modification of CENP-A chromatin or CENP-A/H3 ratios, licensing CENP-A chromatin for reassembly in the next G1 phase (grey arrow “b”)

One possible mechanism would involve a CDK-mediated phosphorylation event, installed before G1, that is essential to "licence" assembly in G1 phase, potentially by creating a PLK1 docking site (Fig. 4a). Intriguingly, Serine 98 in M18BP1 is a target for both CDK1 and PLK1, at least in vitro, and could, conceptually, provide a G2 licensing mark that facilitates PLK1 binding via its POLO-box domain in G1, a possibility also discussed by the authors of this study (Conti et al. 2024). Importantly, in such a scenario, the CDK activity that installed the phosphoresidue is no longer active by the time it triggers its downstream effect, a key element of licensing. Whether such a scenario exists requires a better understanding of the temporal dynamics of CDK1/2 and PLK1 phosphorylation of the assembly machinery.

Interestingly, a Xenopus isoform of M18BP1 localises to centromeres already in metaphase, where it binds to CENP-C in a CDK-dependent manner (French and Straight 2019). This intriguing positive regulation brings a key assembly factor to the centromere and opens the possibility of licensing subsequent assembly in G1 phase.

However, more recent work indicates that the mitotic form of M18BP1 acts as a competitive inhibitor of HJURP binding to CENP-C in a phosphorylation-dependent manner (Flores Servin et al. 2023). Interestingly, mutants in M18BP1 that disrupt mitotic binding also show partial loss of subsequent CENP-A assembly in G1 phase (French and Straight 2019). Thus, it is possible that the mitotic CDK-dependent recruitment of M18BP1, whilst initially inhibitory, acts as a licensing signal for efficient subsequent CENP-A assembly in G1 phase when CDK levels are low.

In human cells, M18BP1 is recruited to centromeres primarily during mitotic exit and G1 phase (Fujita et al. 2007; Dambacher et al. 2012), although a low level of M18BP1 is present at metaphase centromeres (McKinley and Cheeseman 2014). Thus, a form of CDK-mediated licensing of the Mis18 complex in mitosis in human cells may be a possibility. Testing these ideas will require a better understanding of the Mis18 complex binding partners at the CCAN and the spatiotemporal phosphorylation dynamics of these proteins.

Another possible licensing mechanism may involve rendering CENP-A nucleosomes competent for further loading. An intriguing possibility is that the recycling of CENP-A nucleosomes onto sister chromatids during S phase (Jansen et al. 2007) creates a defined mixture of CENP-A and newly loaded H3-containing nucleosomes at the replication fork. It has previously been proposed that the resulting CENP-A/H3 dinucleosome arrangement may provide a unique signature for recognition by the assembly machinery in the next G1 phase (Musacchio and Desai 2017; Pan et al. 2019)(Fig. 4b). However, H3 or H3.3-containing nucleosomes are much more abundant than CENP-A, even at centromeres (Bodor et al. 2014), making it difficult to envisage how a unique CENP-A/H3 arrangement is created only during CENP-A recycling during S phase. Thus, testing this model requires careful measurements of the CENP-A/H3 distribution at centromeres, which, thus far, has been technically challenging.

Alternatively, licensing may constitute a specific modification that is installed onto CENP-A nucleosomes post-assembly in G1, which could serve as a signal for assembly in the next cell cycle (Fig. 4b). This would restrict assembly to sites specified by licensed ancestral nucleosomes. In this context, the identification of H4K20 monomethylation within CENP-A nucleosomes is of particular interest (Hori et al. 2014; Arimura et al. 2019), although it’s modification at CENP-A appears not to be cell cycle regulated.

Furthermore, phosphorylation of CENP-C mediated by CDK1 has been reported to be functionally important for stable binding to CENP-A and kinetochore recruitment in mitosis (Watanabe et al. 2019), which may feed into efficient recruitment of CENP-A in the next cell cycle.

Finally, a few studies have identified possible"maturation"factors implicated in stabilising CENP-A, such as RSF1 and MgcRacGAP (Perpelescu et al. 2009; Lagana et al. 2010), which were proposed to allow CENP-A transmission to the next cell generation. The action of these proteins is still ill-defined, and whether CENP-A becomes a regulated substrate for loading remains speculative.

It is important to note that assembly in G1 phase is not universal. For instance, mitotic Arabidopsis cells, Drosophila germline stem cells and fission yeast assemble CENP-A in G2 phase (Lermontova et al. 2007; Lando et al. 2012; Ranjan et al. 2019; Dattoli et al. 2020). This indicates that while a once-per-cell-cycle mode of assembly may be universal, any licensing mechanism may be wired differently in these organisms or even be plastic during development.

### What maintains CENP-A homeostasis?

**Fig. 5:**
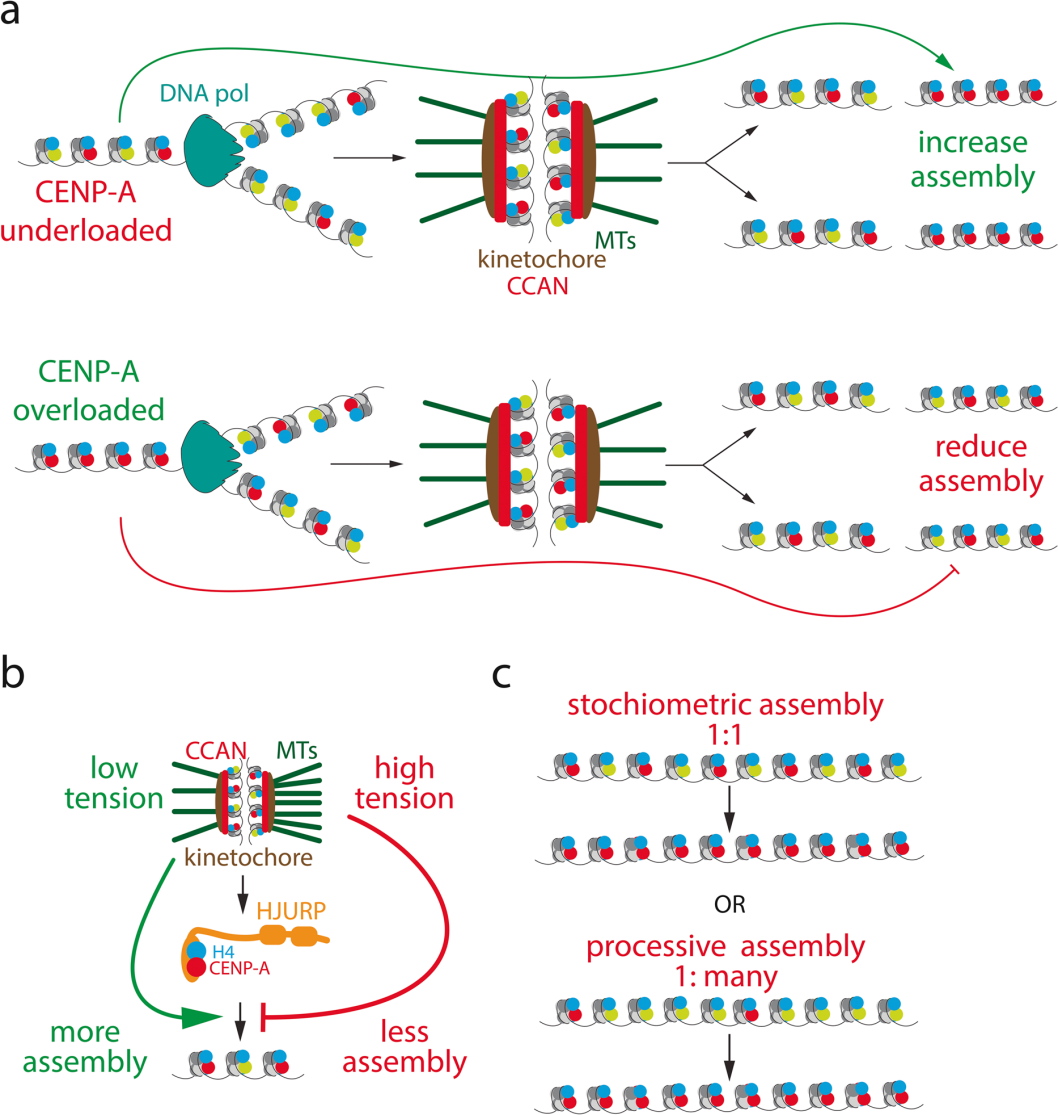
Maintenance of CENP-A homeostasis. **a)** Stochastic variation in the assembly of CENP-A likely requires compensatory mechanisms. Centromeres underloaded in the previous cell cycle may signal to the assembly machinery to increase assembly in the next cycle, while overloaded centromeres inhibit the assembly machinery in the next. **b)** A variation of the model in (a). Here, underloaded or overloaded CENP-A at centromeres translates into weaker and stronger kinetochores and less and more tension across kinetochores and centromeric chromatin, which may serve as a signal to either increase or attenuate assembly in the next G1. **c)** Maintenance of CENP-A homeostasis may require switching from stoichiometric assembly to processive assembly, controlling CENP-A assembly rates

How the correct “amount” of CENP-A is assembled every cell cycle remains an open question. While CENP-A chromatin generally promotes CENP-A assembly, one can envisage homeostatic mechanisms that link the levels of CENP-A chromatin to the strength of assembly in the next cell cycle. In such a scenario, too little CENP-A promotes assembly and too much inhibits the assembly of the next round. This may involve mechanisms where e.g. a limiting “inhibitor” bound by CENP-A chromatin can titrate out the assembly machinery, such that too much CENP-A reduces further assembly (Fig. 5a).

Another elegant early model to explain cell cycle coupling and CENP-A homeostasis proposed that stretching of centromeric chromatin due to spindle forces facilitates recruitment of the loading machinery or stabilisation of CENP-A, marking the stretched chromatin as the active centromere (Mellone and Allshire 2003) (Fig. 5b). The timing of CENP-A assembly in early G1 phase is consistent with such a model. However, allowing cells to transit mitosis in the absence of microtubules (and therefore without chromatin stretching) does not prevent CENP-A assembly (Jansen et al. 2007). While this excludes a strict requirement for chromatin stretching in CENP-A assembly, it remains possible that centromere tension regulates the degree of assembly, coupling centromere strength to its functional output. This idea was further developed by (Brown and Xu 2009) and extended by (Stankovic and Jansen 2017), who hypothesised that the centromere may act as a tensiometer. Here, tension between the two sister chromatids in metaphase negatively regulates the centromere assembly signal, where strong separation of the centromeres translates to a weak assembly signal, and vice versa (Fig. 5b). While this model remains to be tested, it offers an elegant mechanism to maintain centromere strength within functional boundaries.

### Further Open Questions

Key questions remain about how the quantity of CENP-A incorporation is controlled. Does a single ancestral CENP-A nucleosome recruit M18BP1 and HJURP to load a single new CENP-A nucleosome, or can it template the loading of several nearby CENP-A nucleosomes in a processive manner (Fig. 5c)? The variability in the level of CENP-A across centromeres (Bodor et al. 2014) and the observation that CENP-A can overload (Jansen et al. 2007; Bodor et al. 2014) but also be selectively removed (Nechemia-Arbely et al. 2019; Mitra et al. 2020a) suggest that CENP-A loading is not a 1:1 affair, but is dynamically controlled. The molecular basis of this control and understanding what turns off CENP-A assembly remain important open questions.

Once assembled, tuning of CENP-A levels may be controlled by mechanisms that actively turnover CENP-A nucleosomes. CCAN proteins at centromeres are continuously SUMOylated and deSUMOylated, tuning their levels and impacting CENP-A stability (Mitra et al. 2020a, b; van den Berg and Jansen 2023; van den Berg et al. 2023). Thus, the stable transmission of CENP-A nucleosomes appears to be dynamically regulated and responsive to signals that balance centromeric chromatin levels. Furthermore, heterochromatin domains marked by H3K9me3 adjacent to active centromeres serve as boundaries to CENP-A and restrict CENP-A nucleosome position and levels (Carty et al. 2025). Thus, likely multiple pathways converge to maintain CENP-A homeostasis within strict boundaries maintaining mitotic fidelity.

Perhaps the most fundamental unanswered question remains why assembly is restricted to early G1? Possible answers include the requirement to uncouple the assembly of a minority histone, CENP-A from bulk histone assembly in S phase, to prevent interference and competition for common factors such as histone H4 (Valente et al. 2012). Alternatively, the assembly in G1 allows for the reduction of CENP-A to half its complement and mixing of CENP-A with H3 nucleosomes during S phase. A unique CENP-A/H3 chromatin template may form a specific chromatin substrate for kinetochore assembly in mitosis (Jansen et al. 2007; Dunleavy et al. 2011). There is some support for this model, e.g. the observation that CENP-T/W associates with H3-containing nucleosomes, suggesting that H3 adjacent to CENP-A may be architecturally relevant (Hori et al. 2008). However, there is little experimental evidence showing how unscheduled loading negatively affects chromosome segregation or centromere maintenance. Testing these ideas will require more spatiotemporally controlled surgical methods to manipulate the timing of CENP-A assembly without disrupting cell cycle progression.
